# Patients with Cholangiocarcinoma Present Specific RNA Profiles in Serum and Urine Extracellular Vesicles Mirroring the Tumor Expression: Novel Liquid Biopsy Biomarkers for Disease Diagnosis

**DOI:** 10.3390/cells9030721

**Published:** 2020-03-14

**Authors:** Ainhoa Lapitz, Ander Arbelaiz, Colm J. O’Rourke, Jose L. Lavin, Adelaida La Casta, Cesar Ibarra, Juan P. Jimeno, Alvaro Santos-Laso, Laura Izquierdo-Sanchez, Marcin Krawczyk, Maria J. Perugorria, Raul Jimenez-Aguero, Alberto Sanchez-Campos, Ioana Riaño, Esperanza Gónzalez, Frank Lammert, Marco Marzioni, Rocio I.R. Macias, Jose J.G. Marin, Tom H. Karlsen, Luis Bujanda, Juan M. Falcón-Pérez, Jesper B. Andersen, Ana M. Aransay, Pedro M. Rodrigues, Jesus M. Banales

**Affiliations:** 1 Department of Liver and Gastrointestinal Diseases, Biodonostia Health Research Institute, Donostia University Hospital, University of the Basque Country (UPV/EHU), 20014 San Sebastian, Spain; ainhoa.lapitz@biodonostia.org (A.L.); aarbelaizcossio@gmail.com (A.A.); adelaida.lacastamunoa@osakidetza.eus (A.L.C.); alvaro.santos@biodonostia.org (A.S.-L.); laura.izquierdo@biodonostia.org (L.I.-S.); matxus.perugorria@biodonostia.org (M.J.P.); raul.jimenezaguero@osakidetza.eus (R.J.-A.); ioana.rianofernandez@osakidetza.eus (I.R.); luis.bujandafernandezdepierola@osakidetza.eus (L.B.); 2 Department of Health and Medical Sciences, Biotech Research & Innovation Centre (BRIC), 2200 Copenhagen, Denmark; colm.rourke@bric.ku.dk (C.J.O.); jesper.andersen@bric.ku.dk (J.B.A.); 3 CIC bioGUNE, Genome Analysis Platform, 48160 Derio, Spain; joluito@gmail.com (J.L.L.); amaransay@cicbiogune.es (A.M.A.); 4 Hospital of Cruces, 48903 Bilbao, Spain; cesar.ibarraponcedeleon@osakidetza.eus (C.I.); albertosancam@gmail.com (A.S.-C.); 5 “Complejo Hospitalario de Navarra”, 31008 Pamplona, Spain; jp.jimeno.garcia@navarra.es; 6 Carlos III National Institute of Health, Center for the Study of Liver and Gastrointestinal Diseases (CIBERehd), 28220 Madrid, Spain; jfalcon@cicbiogune.es; 7 Department of Medicine II, Saarland University Medical Centre, Saarland University, 66421 Homburg, Germany; Marcin.Krawczyk@uks.eu (M.K.); frank.lammert@uks.eu (F.L.); 8 Department of General, Transplant and Liver Surgery, Laboratory of Metabolic Liver Diseases, Centre for Preclinical Research, 02-091 Warsaw, Poland; 9 Center for Cooperative Research in Biosciences (CIC bioGUNE), Basque Research and Technology Alliance (BRTA), Exosomes Laboratory, 48160 Derio, Spain; egonzalez@cicbiogune.es; 10 Department of Gastroenterology, “Università Politecnica delle Marche”, 60121 Ancona, Italy; m.marzioni@staff.univpm.it; 11 Experimental Hepatology and Drug Targeting (HEVEFARM), Biomedical Research Institute of Salamanca (IBSAL), 37007 Salamanca, Spain; rociorm@usal.es (R.I.R.M.); jjgmarin@usal.es (J.J.G.M.); 12 Division of Cancer Medicine, Surgery and Transplantation, Norwegian PSC Research Center, Oslo University Hospital, 0372 Oslo, Spain; t.h.karlsen@medisin.uio.no; 13 IKERBASQUE, Basque Foundation for Science, 48013 Bilbao, Spain

**Keywords:** biomarkers, cholangiocarcinoma, extracellular vesicles, liquid biopsy, transcriptomics

## Abstract

Cholangiocarcinoma (CCA) comprises a group of heterogeneous biliary cancers with dismal prognosis. The etiologies of most CCAs are unknown, but primary sclerosing cholangitis (PSC) is a risk factor. Non-invasive diagnosis of CCA is challenging and accurate biomarkers are lacking. We aimed to characterize the transcriptomic profile of serum and urine extracellular vesicles (EVs) from patients with CCA, PSC, ulcerative colitis (UC), and healthy individuals. Serum and urine EVs were isolated by serial ultracentrifugations and characterized by nanoparticle tracking analysis, transmission electron microscopy, and immunoblotting. EVs transcriptome was determined by *Illumina* gene expression array [messenger RNAs (mRNA) and non-coding RNAs (ncRNAs)]. Differential RNA profiles were found in serum and urine EVs from patients with CCA compared to control groups (disease and healthy), showing high diagnostic capacity. The comparison of the mRNA profiles of serum or urine EVs from patients with CCA with the transcriptome of tumor tissues from two cohorts of patients, CCA cells in vitro, and CCA cells-derived EVs, identified 105 and 39 commonly-altered transcripts, respectively. Gene ontology analysis indicated that most commonly-altered mRNAs participate in carcinogenic steps. Overall, patients with CCA present specific RNA profiles in EVs mirroring the tumor, and constituting novel promising liquid biopsy biomarkers.

## 1. Introduction

Cholangiocarcinomas (CCAs) are heterogeneous biliary malignancies characterized by dismal prognosis. The incidence and mortality rates of these cancers are rapidly increasing globally, currently accounting for ~15% of all primary liver cancers and ~3% of gastrointestinal malignancies [1,2,3]. According to their anatomical localization, CCAs are classified into intrahepatic (iCCA), perihilar (pCCA), or distal (dCCA). The etiology of most CCAs is unknown. However, several risk factors have been described, including the presence of primary sclerosing cholangitis (PSC: 5–15% develops CCA), a chronic cholestatic liver disease that is associated with autoimmune phenomena against the intra- and extrahepatic bile ducts [1,4,5]. Importantly, 70–80% of patients with PSC concomitantly present inflammatory bowel disease (IBD), mainly ulcerative colitis (UC), which is thought to precede the development of the liver disease [4].

CCAs are generally asymptomatic in early stages, being therefore commonly diagnosed in advanced phases when the disease is disseminated. Late diagnosis combined with the chemoresistant nature of these tumors [6] highly compromise the current therapeutic options, mainly based on surgery, significantly impacting on patient’s welfare and outcome [1,2]. The diagnosis of CCA is usually conducted by combining clinical, biochemical, radiological, and histological information. Imaging techniques usually rely on computed tomography (CT), magnetic resonance imaging (MRI), magnetic resonance cholangiopancreatography (MRCP), positron emission tomography (PET), percutaneous transhepatic cholangiography (PTC), endoscopic retrograde cholangiopancreatography (ERCP), or endoscopic ultrasound depending on the tumor location [1,5,7]. However, imaging proceedings have important limitations, as they are not accurate enough to determine the malignity of the tumor masses, particularly in early stages, as well as to differentiate between the main primary liver cancers, i.e., iCCA, hepatocellular carcinoma (HCC) or HCC-CCA mixed tumors, which is fundamental to provide the appropriate standards of care. On the other hand, MRCP and histological analysis (biopsy or brushing cytology) comprise the major diagnostic tools for PSC [4,8,9]. In addition, the measurement of non-specific serum tumor biomarkers [i.e., carbohydrate antigen 19-9 (CA19-9) and carcinoembryonic antigen (CEA)] is commonly conducted in order to help in the diagnosis of CCA, but their low sensitivity (particularly in early stages of the disease) and specificity (also elevated in some PSC patients without cancer), raise important concerns regarding their clinical utility [7,10]. Therefore, tumor biopsy is currently mandatory to confirm the diagnosis and staging of CCA, guiding the clinical management of these patients [11]. Based on all of these diagnostic concerns, there is an urgent need to determine new accurate, non-invasive biomarkers for the early diagnosis of CCA, particularly in patients at risk.

In the last decade, extracellular vesicles (EVs) have been envisioned as promising tools in the quest for tumor biomarkers and as important mediators of disease pathogenesis [12]. EVs constitute a heterogeneous population of lipid bilayered spheres (30 nm – 2 μm in diameter) containing diverse biomolecules (e.g., proteins, nucleic acids, lipids, and metabolites), which are released by cells and found in all biofluids (e.g., blood and urine) [13,14]. Taking into account their biogenesis, EVs may be classified as exosomes, microvesicles (MV), and apoptotic bodies. These small vesicles participate in cell-to-cell communications, modulating signaling pathways in pathobiology [13,14,15,16,17]. We previously reported a differential proteomic profile of serum EVs from patients with CCA, HCC, or PSC, as well as from healthy individuals, identifying accurate candidate biomarkers for the differential diagnosis of these diseases [18]. Considering that tumor cells can also release RNAs encapsulated within EVs, and that their profiles can mimic the cellular state/alterations, an extensive characterization of the RNA content from serum and urine EVs from patients with CCA (and control conditions) might provide new diagnostic biomarkers as well as therapeutic targets.

In this study, we aimed to characterize the RNA profile of serum and urine EVs from patients with CCA, PSC, or UC, as well as healthy individuals, and identify candidate diagnostic biomarkers mirroring their tumor cell expression within the liquid biopsy concept. For this purpose, the expression of selected candidates was evaluated in human CCA tumor and surrounding healthy tissues from two independent cohorts of patients [The Cancer Genome Atlas (TCGA) and Copenhagen], as well as in cell cultures (CCA vs. normal) and EVs released by normal or tumor human cholangiocytes in vitro.

## 2. Materials and Methods

### 2.1. Patients

Serum and urine samples from patients with CCA (n = 12 and 23, respectively), PSC (n = 6 and 5, respectively), UC (n = 8 and 12, respectively), and healthy individuals (n = 9 and 5, respectively) were obtained from Donostia University Hospital (San Sebastian, Spain), Cruces University Hospital (Bilbao, Spain), and “Complejo Hospitalario de Navarra” (Pamplona, Spain). The Ethical Committees for Clinical Research from each participating institution approved all the research protocols and all patients accepted to participate in the study and signed the written consents to allow the use of their samples for biomedical research. Clinical characteristics of patients and tumors are summarized in Supplementary Table S1. The diagnosis of PSC was based on the European Association for the Study of the Liver (EASL) guidelines [19] by demonstrating the presence of bile duct alterations (strictures or irregularities in intrahepatic and extrahepatic bile ducts) using MRCP after excluding secondary causes of cholangitis. The diagnosis of UC was performed by combining endoscopic and histological studies, mainly colonoscopy in parallel with hematoxylin and eosin (H&E) staining, after excluding other potential diseases. Finally, CCA diagnosis was confirmed by histological analysis of tumor samples and/or through the combination of clinical, biochemical and radiological approaches. Tumor stage was determined based on the 7^th^ edition of the American Joint Committee on Cancer (AJCC) classification.

RNA-seq data from the TCGA cohort (36 CCAs and 9 surrounding liver samples) [20], downloaded as level 3 data through FireBrowse portal [BROAD Institute of MIT & Harvard, MA, USA (source: https://gdac.broadinstitute.org/)], and whole transcriptome profiling [Human Transcriptome (HT) BeadChips (Illumina Inc., San Diego, CA, USA)] of the “Copenhagen cohort” including 217 CCA surgical specimens (153 iCCA, 43 pCCA, 15 dCCA, 6 unknown location), 143 normal surrounding liver samples, and 9 normal intrahepatic bile ducts (GSE26566) [21,22] were used to evaluate the expression of serum and urine biomarkers in tumor tissue.

### 2.2. Cell Cultures

Normal human cholangiocytes (NHCs) were isolated from normal liver tissue and characterized as previously described [23,24,25]. Furthermore, two commercial human CCA cell lines (EGI1 and TFK1, Leibniz Institute DSMZ-German Collection of Microoganism and Cell Cultures, Germany) were used. NHC and EGI1 cells were cultured in fully-supplemented DMEM/F-12 medium, as previously described [23,24,25], while TFK1 cells were cultured in DMEM/F-12 supplemented with 10% fetal bovine serum (FBS; Gibco, Thermo Fisher Scientific, Waltham, MA, USA) and 1% penicillin/streptomycin (P/S; Gibco). Cells were seeded in 150 mm collagen-coated tissue culture dishes (4 × 10^6^ cells) with each respective cell culture medium and left for plate attachment overnight. Afterwards, cells were washed with phosphate-buffered saline (PBS) and incubated with “EV recollection media” (DMEM/F-12+Glutamax supplemented with 1% P/S, and without serum). After 48 h, cells were harvested for RNA isolation and cell culture media was collected and stored at −80 °C for subsequent EVs isolation. Cells were grown at 37 °C in a humidified chamber of 5% CO_2_. During all the experiments, mycoplasm test was performed by conventional PCR and cells were tested as mycoplasm negative.

### 2.3. Isolation of EVs from Serum, Urine, and Cell Cultures

Serum, urine, and cell-derived EVs were isolated as previously described [18]. Briefly, 1 mL of serum, 50 mL of urine, or 300 mL of cell culture media (frozen at −80 °C) were thawed at room temperature and further processed through serial differential ultracentrifugation steps at 4 °C. First, in order to remove cell debris, serum, urine and cell culture media were centrifuged at 10,000× *g* for 30 min and subsequently ultracentrifuged at 100,000× *g* for 75 min, to pellet the EVs, which were then washed with PBS and pelleted again after ultracentrifugation at 100,000× *g* for 75 min. Finally, the pelleted EV fraction was resuspended in 20 μL of PBS and then stored at −80 °C for further analysis.

### 2.4. Transmission Electron Microscopy (TEM)

For the characterization of EVs, the isolated fraction of EVs was stained negatively and analyzed by TEM. EV samples were directly adsorbed onto glow-discharged (60 seg low discharging using a PELCO easy-glow device) carbon-coated copper grid (300 mesh). Afterwards, grids were fixed with 2% paraformaldehyde (PFA) in phosphate buffer (PB 0.2M pH 7.4) for 20 min and washed with distilled water. Then, the contrast staining was made by incubating the grids with 4% uranyl acetate (UA) at 4 °C for 15 min. TEM images were obtained by using TECNAI G2 20 C-TWIN high-resolution transmission electron microscope, at an acceleration voltage of 200 kV.

### 2.5. Immunoblotting

Protein levels of both EV and endoplasmic reticulum markers (i.e., CD63 and CD81 vs. GRP78, respectively) were evaluated in serum and urine EVs and in whole-cell extracts (WCEs) by immunobloting. Total protein concentration was calculated with the Micro BCA protein assay kit (Thermo Fisher Scientific,), following the manufacturer’s instructions. Loading buffer [50 mM Tris-HCl, 2% SDS, 10% glycerol and 0.1% bromophenol blue, without β-mercaptoethanol or dithiothreitol (DTT)] was added to protein samples, followed by heat denaturation at 95 °C for 5 min. Then, 10 and 4 μg of total protein from serum and urine EVs, respectively, were separated by 12.5% sodium dodecyl sulfate-polyacrilamide gel electrophoresis (SDS-PAGE) and electro-transferred onto a nitrocellulose membrane (GE Healthcare, Chicago, IL, USA) and blocked with 5% skim milk powder/tris-buffered saline (TBS)-0.1% tween (TBS-Tween) for 1 h. Afterwards, membranes were probed overnight at 4°C with the appropriate primary antibodies [anti-CD81 (BD Biosciences), anti-CD63 (DSHB), and anti-GRP78 (BD Biosciences, San Jose, CA, USA)] at 1:500 dilution in blocking solution and, after three washes with TBS-Tween (5 min each), horseradish peroxidase-conjugated secondary antibody (anti-mouse; Cell Signaling, Danvers, MA, USA) at a dilution of 1:5000 (in milk blocking solution) were incubated for 1 h at room temperature. Membranes were developed for protein detection using ECL plus (Thermo Fisher Scientific), with the iBright FL1500 Western Blot Imaging System (Thermo Fisher Scientific).

### 2.6. EV Size and Concentration

Size distribution and concentration of EVs were evaluated by nanoparticle tracking analysis (NTA) using a NanoSight LM10 System (Malvern, UK) further equipped with fast video capture and a particle-tracking software. NTA post-acquisition settings were kept constant for all samples. Each video was analyzed for obtaining the mean and mode vesicle size as well as particle concentration.

### 2.7. Total RNA Isolation

After EVs isolation, total RNA was extracted using the miRCURY™ RNA Isolation Kit (Qiagen, Hilden, Germany) following manufacturer’s specifications. Afterwards, total RNA was resuspended in 20 μL of distilled H_2_O and later used for transcriptomic analysis. Regarding cell samples, total RNA was extracted using the TRIzol^®^ reagent according to the manufacturer’s instructions (Life Technologies Corp., Carlsbad, CA, USA).

### 2.8. Illumina Gene Expression Array

Illumina HumanHT-12 WG-DASL V4.0 R2 expression beadchips were used to characterize gene expression [messenger RNAs (mRNAs) and non-coding RNAs (ncRNAs)]. The quality of RNA samples was measured using a RNA Pico Chip Bioanalyzer (Agilent Technologies, Santa Clara, CA, USA). 200 ng of RNA samples were used for the array. The cDNA synthesis, prequalification, amplification, labeling and hybridization of the samples were performed following the WG-DASL HT Assay Lab protocol (Illumina Inc.). The amplified cDNAs were hybridized to the diverse gene-probes of the array and gene expression levels were detected by a HiScan scanner (Illumina Inc.). Raw data were extracted with GenomeStudio analysis software (Illumina Inc.), in the form of GenomeStudio’s Final Report. Raw expression data were background-corrected, log_2_-transformed and quantile-normalized using the lumi R package [26] (Bioconductor repository, Chicago, IL, USA). To perform the Venn diagrams, all the transcripts identified in at least one sample with a “detection *p*-value” < 0.01 were selected. Afterwards, in the comparisons between groups, transcripts that were significantly identified in at least 20% of the samples (with a bilateral *p*-value < 0.05; independent samples two-tailed t-test, not assuming equal variances) were considered for subsequent analysis.

### 2.9. Functional Enrichment Analysis

Functional analysis of candidate liquid biopsy RNA biomarkers was determined by gene ontology (GO) enrichment of biological processes, molecular pathways and functions, by using the Functional Enrichment analysis tool (FunRich) version 3.1.3 (Funrich Industrial Co. Ltd, Hong Kong) [27,28].

### 2.10. Statistical Analysis

Statistical analysis was performed using GraphPad Prism version 6.0 (GraphPad Software, San Diego, CA, USA). Data are shown as boxes and whiskers (min to max). When comparing two groups, non-parametric Mann-Whitney or parametric t-Student tests were conducted. For comparisons between more than two groups, non-parametric Kruskal-Wallis test followed by a posteriori Dunns test of the parametric one-way analysis of variance (ANOVA) test followed by a posteriori Tukey’s post hoc test were used. In order to calculate the diagnostic values of serum and urine RNA biomarkers, allowing to discriminate between patients with CCA, PSC and UC, and healthy individuals, area under the receiver operating characteristic curve (AUC) values were determined using the *SPSS* 20.0 software (IBM, Ehningen, Germany), followed by the calculation of sensitivity (SEN) and specificity (SPE) values, positive predictive value (PPV), negative predictive value (NPV), positive likelihood ratio (PLR), negative likelihood ratio (NLR), and accuracy index (AI). Differences were considered significant when *p* < 0.05. 

## 3. Results

### 3.1. Characterization of Serum and Urine EVs from Patients with CCA, PSC, or UC, and Healthy Individuals

**Figure 1:**
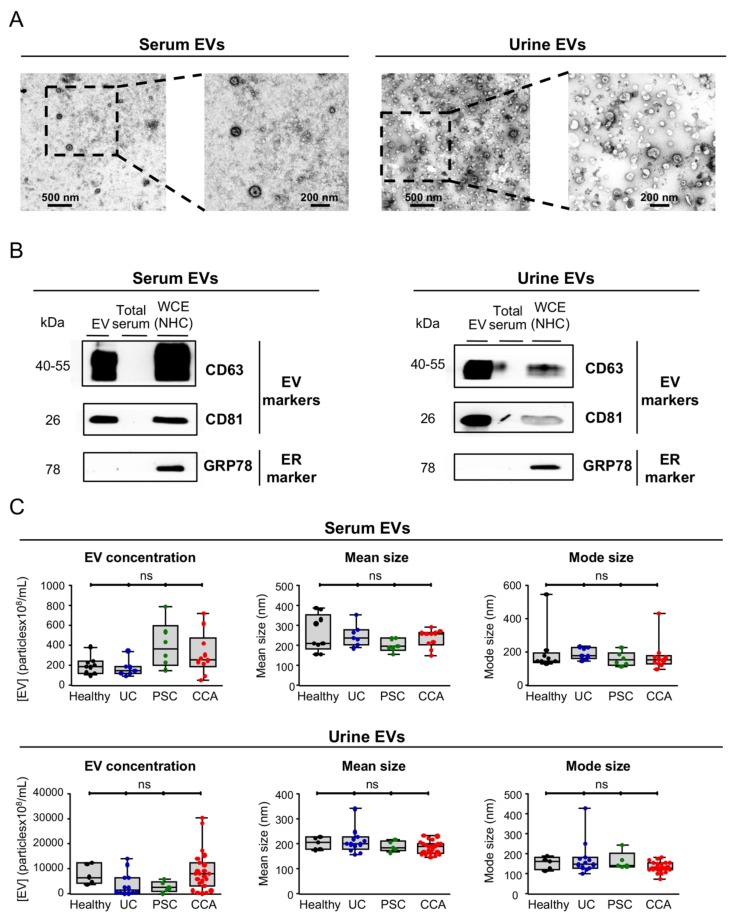
Characterization of serum and urine EVs from patients with CCA, PSC, UC, and healthy controls. In order to validate the protocol for EVs isolation, we used blood serum and urine from healthy individuals. (**A**) TEM images of blood serum (left) and urine (right) EVs from healthy individuals showcasing the typical round shape (~150 nm) and morphology. (**B**) Representative immunoblots of the EV markers CD63 and CD81 (positive controls) and GRP78 (negative control) from EVs isolated from serum (left) and urine (right) of healthy individuals that indicate an enrichment of EV markers and a complete absence of the endoplasmic reticulum (ER) marker GRP78, compared to total serum and whole cell extracts (WCEs) of normal human cholangiocytes (NHC). (**C**) Nanoparticle tracking analysis (NTA) of serum (up) and urine (down) EVs revealing no differences in EV concentration between CCA, PSC, UC, and healthy individuals and a similar EV mode (~180 nm).

After isolation, serum and urine EVs were characterized by TEM, immunoblotting and NTA. In resemblance with our previous findings using the same isolation protocol [18], TEM images showed a typical rounded morphology in the isolated vesicles from both serum and urine (~100–200 nm), corresponding to exosomes and/or small microvesicles (Figure 1A). By immunoblotting, the EV protein markers CD63 and CD81 were highly enriched in the isolated EV fraction, when compared to total serum or NHC whole-cell extracts (WCE), while the endoplasmic reticulum marker 78 kDa glucose-regulated protein (GRP78) was completely absent in isolated serum and urine EVs but only found expressed in WCE from NHCs (Figure 1B), substantiating a proper isolation and a high purity of the obtained EVs. Regarding the size of EVs, NTA revealed no significant differences in the size of serum and urine EVs among groups, presenting an average size of ~180 nm, in resemblance with serum and urine EV concentration, which was found similar in the study population (Figure 1C).

### 3.2. Differential RNA Profiles of Serum EVs from CCA, PSC, UC, and Healthy Individuals

**Figure 2:**
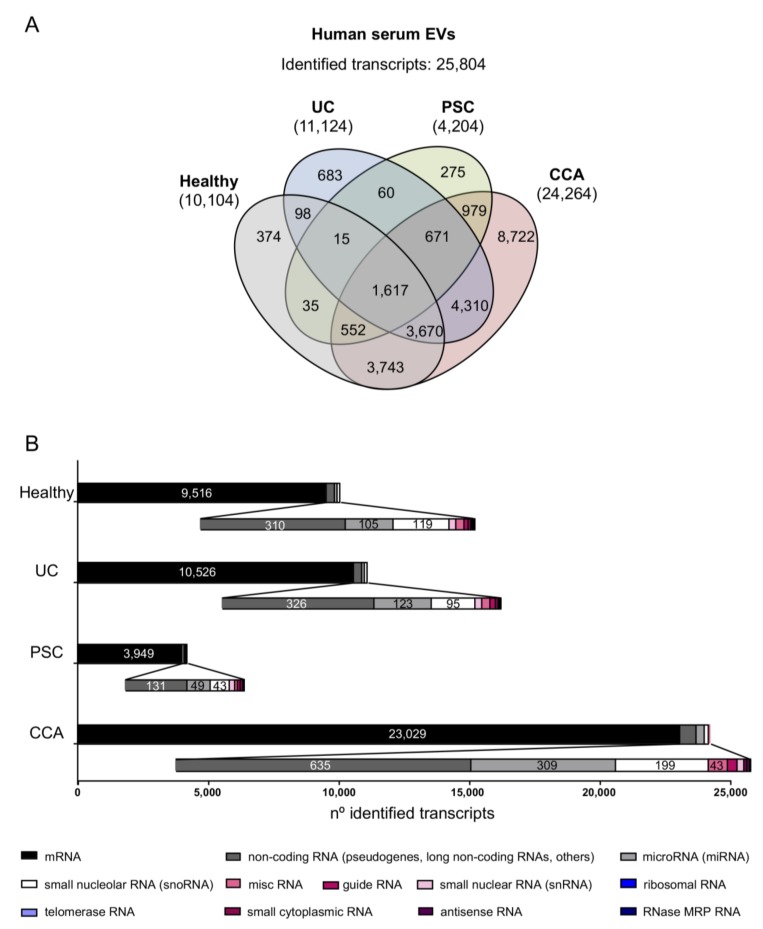
Comparative transcriptomic analysis of serum EVs from patients with CCA, PSC, or UC, and healthy individuals. (**A**) Venn diagrams showing the number of transcripts identified per group. Venn diagrams were generated using InteractiVenn web-based tool [29]. (**B**) Number of transcripts identified within each study group, subclassified according to their type [messenger RNA (mRNA), non-coding RNA (including mostly pseudogenes and long non-coding RNAs, among others), miscelaneous RNA (miscRNA), guide RNA, microRNA (miRNA), small nucleolar RNA (snoRNA), small nuclear RNA (snRNA), ribosomal RNA, telomerase RNA, small cytoplasmic RNA, antisense RNA and RNase MRP RNA]. In all groups, mRNAs constitute the most abundantly identified RNAs, followed by non-coding RNAs (pseudogenes, lncRNAs, and others), miRNAs, and snoRNAs.

**Figure 3:**
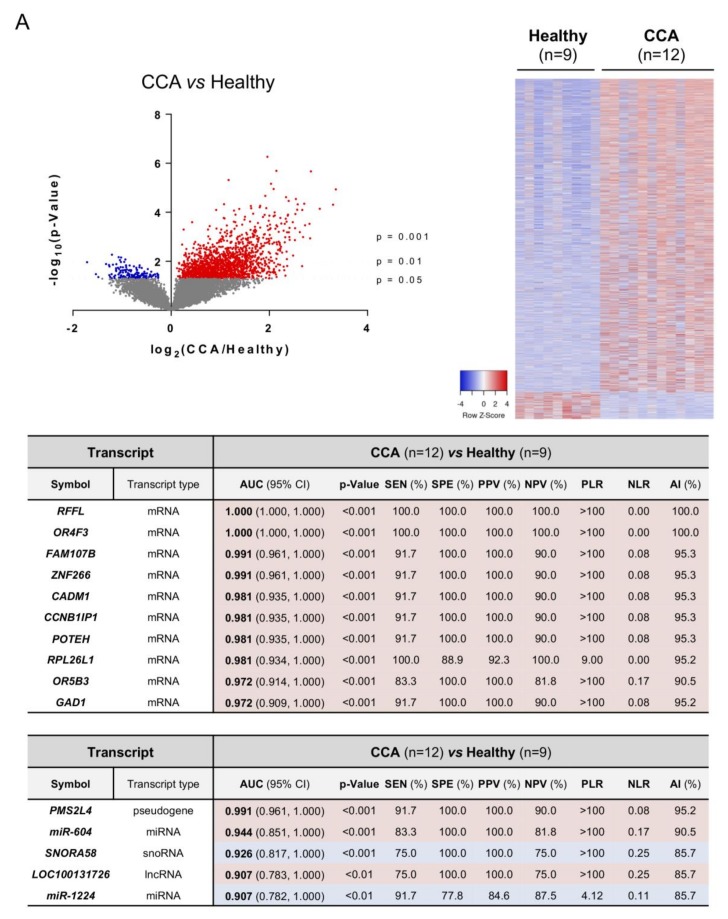

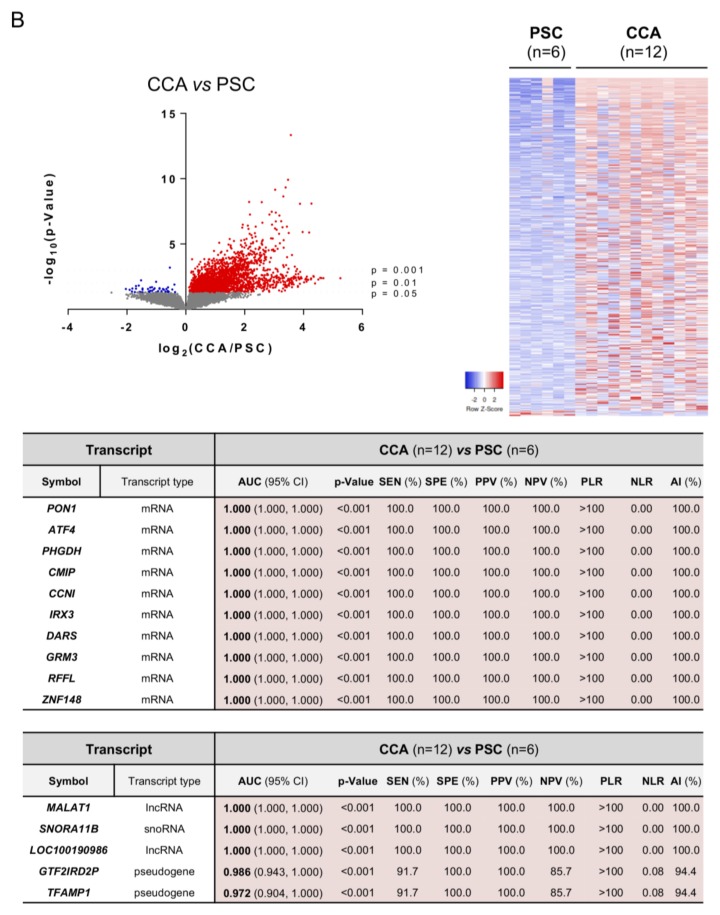

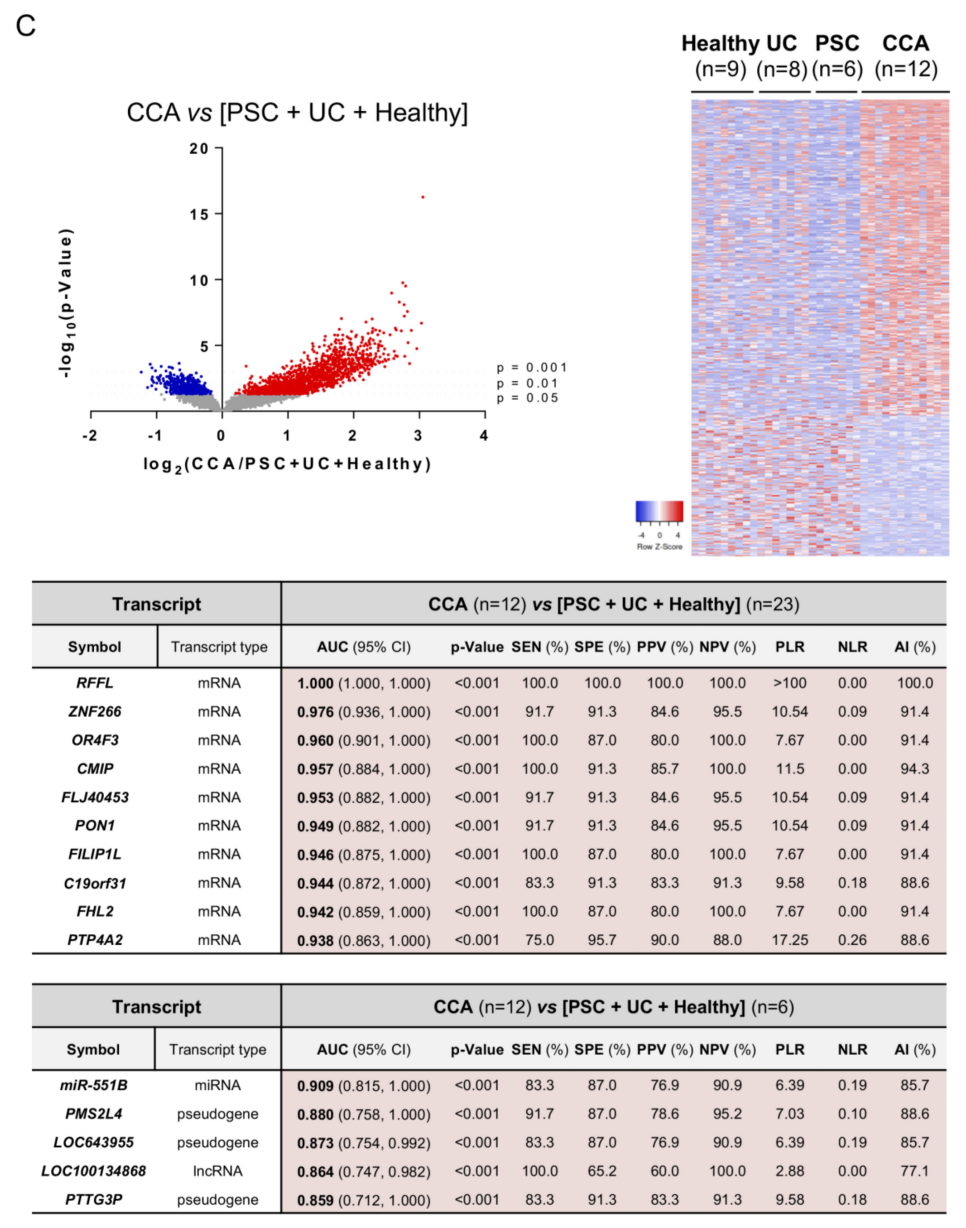
Differential transcriptomic profile of serum EVs and diagnostic capacity. Volcano plot (−log_10_(*p*-value) and log_2_(fold-change); up left), heatmap of the differentially expressed transcripts (up right) and diagrams with the diagnostic capacity with the highest AUC values of the 10 selected mRNAs and 5 selected non-coding RNAs in serum EVs from (**A**) CCA vs. Healthy individuals; (**B**) CCA vs. PSC; (**C**) CCA vs. (PSC + UC + Healthy individuals). Abbreviations: AI, accuracy index; AUC, area under the receiver operating characteristic curve; CI, confidence interval; lncRNA, long non-coding RNA; miRNA, microRNA; NLR, negative likelihood ratio; NPV, negative predictive value; PLR, positive likelihood ratio; PPV, positive predictive value; SEN, sensitivity; snoRNA, small nucleolar RNA; SPE, specificity.

The transcriptomic profiles of serum and urine EVs isolated from patients with CCA, PSC, UC, and healthy controls were determined by RNA microarray-based transcriptomics (Illumina Inc.). Transcriptomic data are available in GSE144521. Considering all the transcripts that were identified in at least one sample included in any of the study groups (detection *p*-value < 0.01), a total of 25,084 transcripts were identified in serum EVs. Among them, 10,104 transcripts were identified in serum EVs isolated from healthy individuals, in parallel with the identification of 11,124, 4204, and 24,264 transcripts in serum EVs from patients with UC, PSC, and CCA, respectively, with 1617 of the identified transcripts being shared among all groups (Figure 2A). In all the study groups, the great majority of the identified transcripts were mRNAs (9516, 10,526, 3949, and 23,029 transcripts found in healthy individuals and patients with UC, PSC, or CCA, respectively), followed by non-coding RNAs such as non-coding RNAs (mainly including pseudogenes, long non-coding RNAs (lncRNAs), among others), microRNAs (miRNAs or miRs), and small nucleolar RNAs (snoRNAs) (Figure 2B; Supplementary Table S2). Other types of RNAs, including small nuclear, miscellaneous, guide, small cytoplasmic, antisense, RNase MRP, ribosomal, and telomerase RNAs were also detected. Next, the transcriptome of serum EVs from the four study groups was determined and compared. Specifically, 1932 transcripts were differentially identified in CCA vs. healthy individuals, 2888 in CCA vs. PSC, and 2807 in CCA vs. “PSC, UC, and healthy individuals” combined as one unique control (disease and healthy) group (Figure 3). Meanwhile, 866 transcripts were differentially identified in serum EVs from patients with PSC compared with a group comprised of patients with UC and healthy individuals (Supplementary Figure S1).

The analysis of candidate RNA biomarkers in serum EVs from CCA vs. healthy individuals pointed out ring finger and FYVE like domain containing E3 ubiquitin protein ligase (RFFL), olfactory receptor family 4 subfamily F member 3 (OR4F3), and the family with sequence similarity 107 member B (FAM107B) as the mRNAs with the highest diagnostic capacity, presenting AUC values of 1.00, 1.00, and 0.991, respectively, along with the non-coding RNAs PMS1 homolog 2 mismatch repair system component pseudogene 4 (PMS2L4), miR-604, and SNORA58 (AUC: 0.991, 0.944, and 0.926, respectively) (Figure 3A). Since PSC is a well-known risk factor that increases the odds of developing CCA, the transcriptomic profiles of serum EVs from patients with CCA vs. PSC were also compared. In particular, the mRNA transcripts paraoxonase 1 (PON1), activating transcription factor 4 (ATF4), and phosphoglycerate dehydrogenase (PHGDH) stood out as the best candidate biomarkers for the differential diagnosis of CCA and PSC, all with AUC values of 1.00 (Figure 3B). Similarly, the lncRNAs metastasis associated lung adenocarcinoma transcript 1 (MALAT1) and LOC100190986, and the snoRNA SNORA11B (AUCs: 1.00) also presented a high accuracy for the identification of CCA vs. PSC (Figure 3B). Of note, several mRNA and non-coding RNAs provided excellent diagnostic values (AUC values up to 0.931 and 0.902, respectively) for the diagnosis of PSC, when compared with patients with UC and healthy individuals (Supplementary Figure S1). Finally, general CCA transcript biomarkers (compared to PSC, UC and healthy individuals combined as one unique control group) were also identified and RFFL, zinc finger protein 266 (ZNF266) and OR4F3 constituted the mRNA transcripts with the highest AUC values (1.00, 0.976, and 0.960, respectively) while miR-551B, PMS2L4, and LOC643955 were the ncRNAs presenting the highest diagnostic capacity, displaying AUC values of 0.909, 0.880, and 0.873, respectively (Figure 3C).

### 3.3. Selective mRNAs Present in Serum EVs from Patients with CCA Mirror Their Levels in Human Tumor Tissue, CCA Cells In Vitro and EVs-Derived from Tumor Cholangiocytes

**Figure 4:**
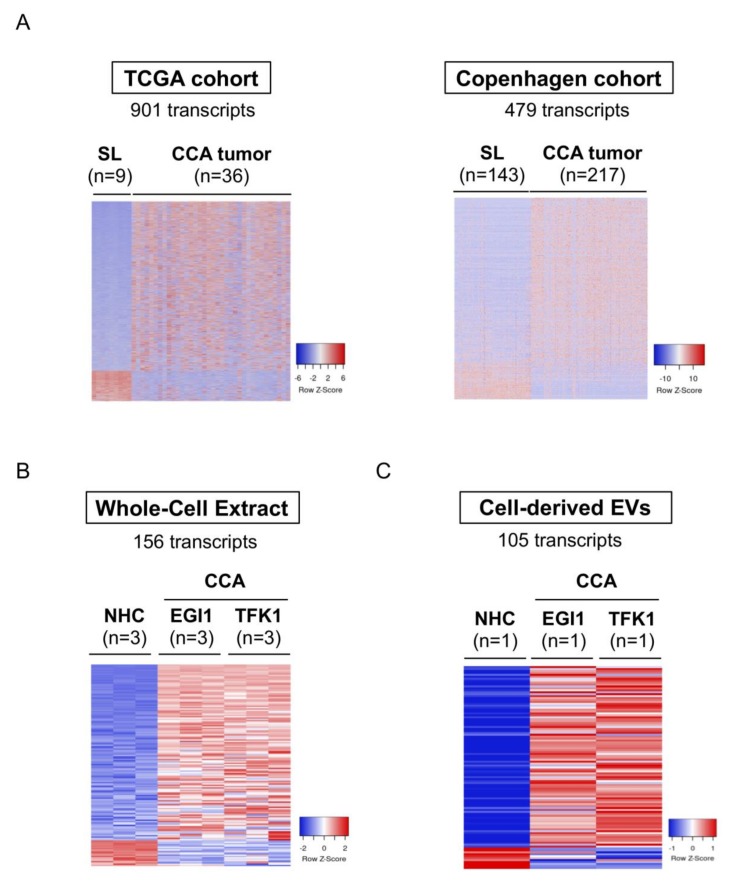

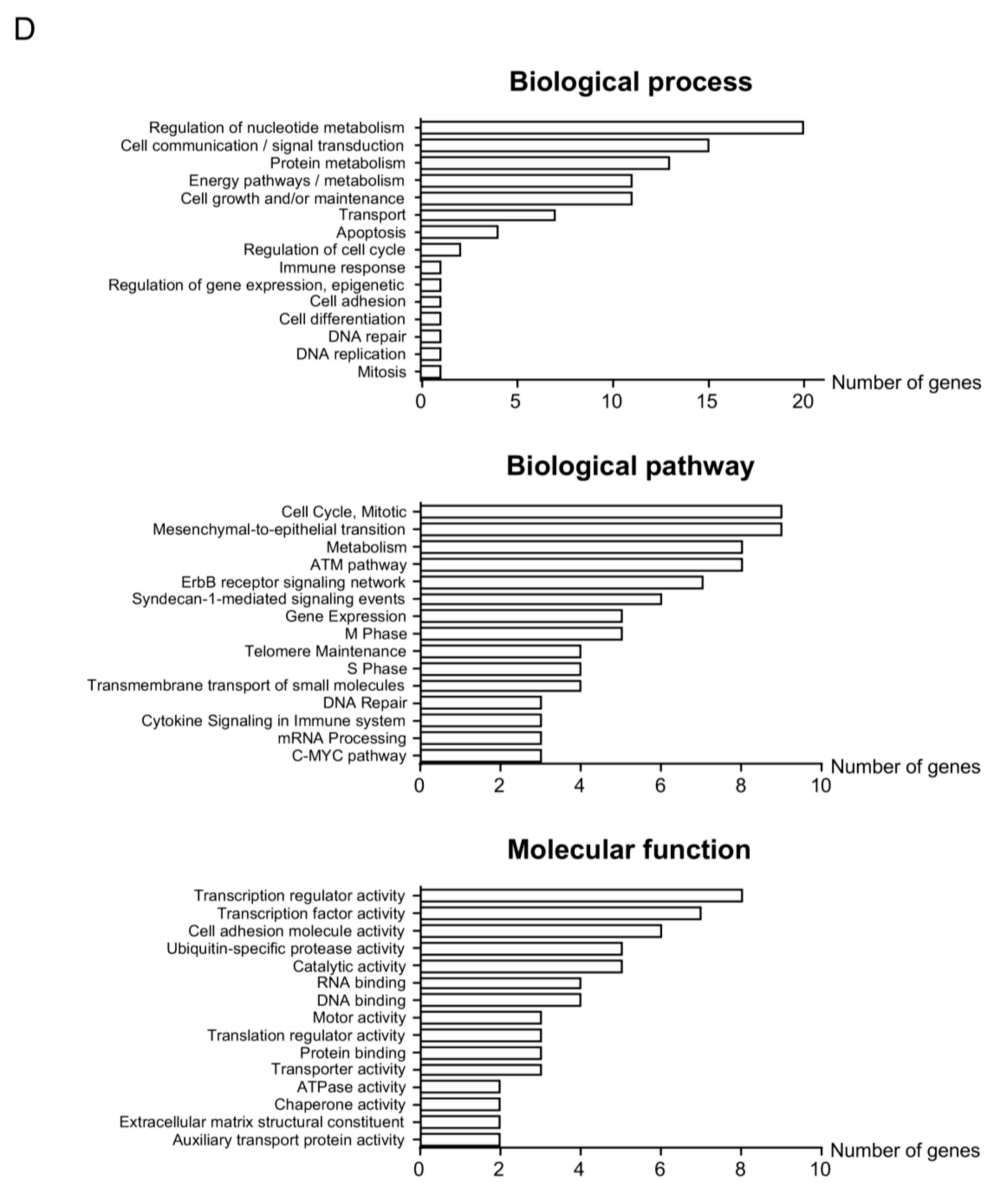
mRNAs commonly deregulated between serum EVs, CCA tumors from two independent cohorts of patients, tumor cells in vitro and in CCA-derived EVs. mRNAs differentially abundant in serum EVs from patients with CCA vs. (PSC + UC + Healthy individuals) were compared with the transcriptome of: (i) patients with CCA from TCGA (n = 36) and “Copenhagen” (n = 217) cohorts, (ii) CCA cells (EGI1 and TFK1) and cell-derived EVs compared to their respective control groups, further selecting the ones that are commonly expressed. Heatmap of the differentially expressed transcripts in (**A**) TCGA (left) and “Copenhagen” cohorts (right); (**B**) Whole-cell extracts from CCA cells and NHCs; (**C**) Cell-derived EVs; (**D**) Gene ontology (GO: FunRich database [27]) analysis of the 105 transcripts commonly altered in serum EVs, CCA human tumors, CCA cells and in cell-derived EVs, highlighting the biological processes and pathways in which the identified transcripts are involved, as well as their biological function. Abbreviations: CCA, cholangiocarcinoma, EVs, extracellular vesicles; NHCs, normal human cholangiocytes; SL, surrounding liver; TCGA, The cancer genome atlas.

After identifying 2807 transcripts significantly altered in serum EVs from patients with CCA compared to patients with PSC, UC, and healthy individuals, we evaluated if the expression of these transcripts were also significantly changed in human CCA tissue, compared to non-tumor surrounding tissue, in two independent international cohorts of patients (TCGA and the “Copenhagen” cohorts). Importantly, 901 out of the 2807 selective RNA transcripts were also altered in the TCGA cohort, presenting the same trend of expression when compared to serum EVs, with 765 transcripts being upregulated while 136 transcripts were downregulated in comparison to non-tumor tissue (Figure 4A, left). These 765 transcripts were then cross-validated in the Copenhagen cohort, in which we were able to identify 479 shared transcripts with the same expression tendency, with 391 being upregulated and 88 reduced when compared with surrounding liver tissue (Figure 4A, right). After selecting the common mRNAs that share the same trend of expression in serum EVs and tumor tissue of patients with CCA compared to controls, we next evaluated their expression levels in two human CCA cell lines (EGI1 and TFK1) compared to NHCs in vitro, obtaining 156 commonly altered transcripts (Figure 4B). Finally, we isolated EVs from these two CCA cell lines and from NHCs and, after their characterization (Supplementary Figure S2) [18], we evaluated their transcriptomic content and cross-validated the previous candidate transcripts, resulting in 105 mRNAs with shared altered levels in serum EVs, tumor tissue, CCA cell lines, and in CCA-derived EVs (Figure 4C).

**Figure 5:**
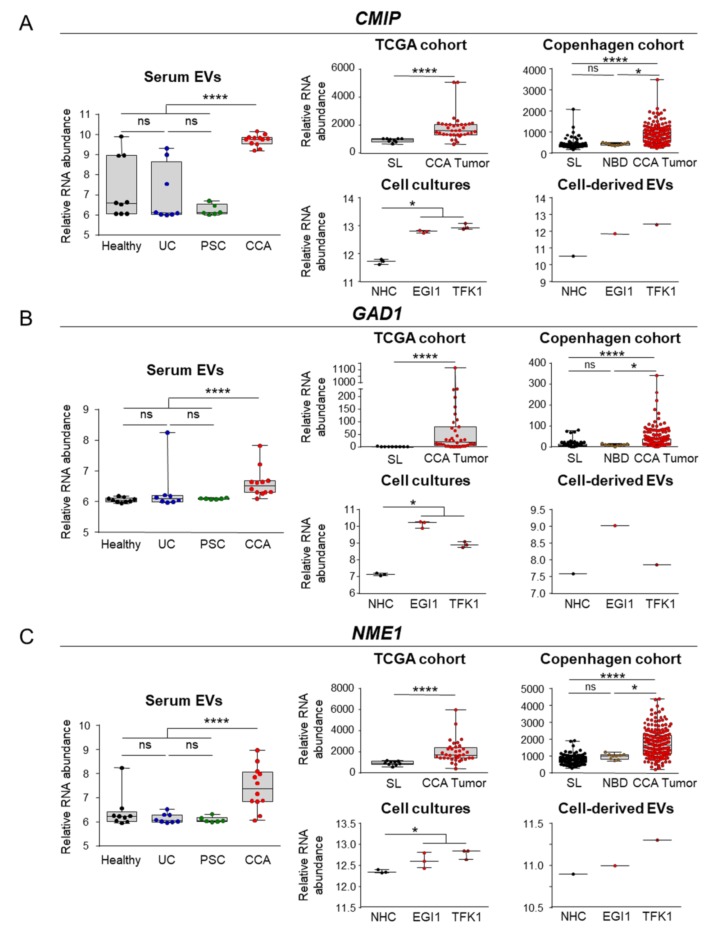

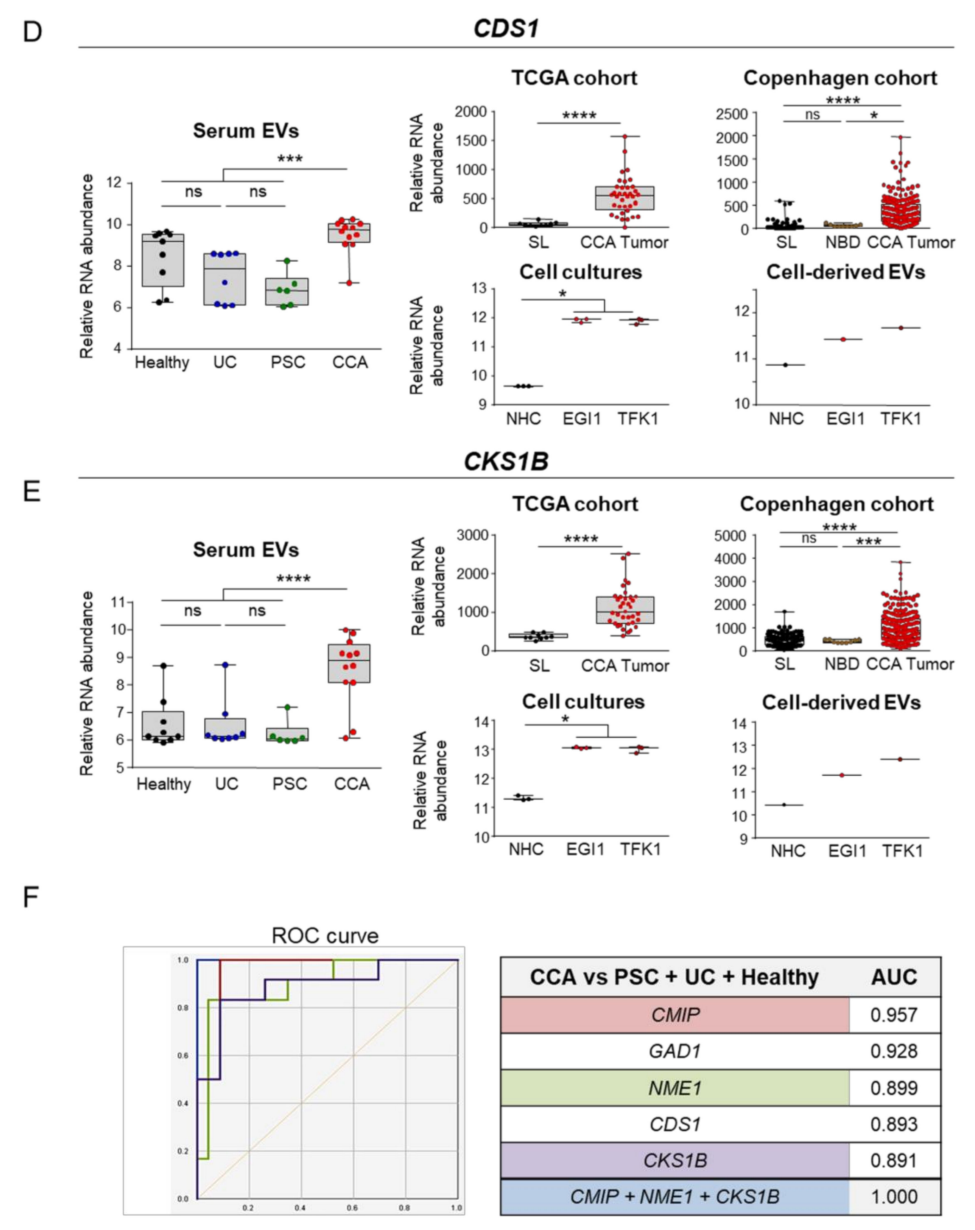
Selected liquid biopsy biomarkers for CCA. From the 105 mRNAs commonly altered in serum EVs, CCA human tumors, CCA cells and in cell-derived EVs compared to their corresponding controls, 5 biomarkers were selected based on their diagnostic capacity. Box plot diagrams with the mRNAs abundance in serum EVs (left), CCA tumors from the TCGA, and “Copenhagen” cohorts, CCA cells and cell-derived EVs (right), compared to their respective controls, for (**A**) *c-Maf inducing protein* (*CMIP*), (**B**) *glutamate decarboxylase 1 (GAD1)*, and (**C**) *NME/NM23 nucleoside diphosphate kinase 1 (NME1)*; (**D**) *CDP-diacylglycerol synthase 1 (CDS1)*, and (**E**) *CDC28 protein kinase regulatory subunit 1B (CKS1B)*. (**F**) Diagnostic prediction (ROC curves and AUC values) of the selected serum liquid biopsy biomarkers and for the combination of CMIP, NME1 and CKS1B for the diagnosis of CCA in comparison with (PSC + UC + Healthy individuals). Abbreviations: AUC, area under the receiver operating characteristic (ROC) curve; EVs, extracellular vesicles; NHC, normal human cholangiocyte; NBD, normal bile ducts; SL, surrounding liver; TCGA, The cancer genome atlas.

Gene ontology (GO) analysis revealed that the 105 mRNAs previously identified code for proteins mainly involved in pivotal processes during carcinogenesis. Specifically, metabolic pathways (nucleic acid and protein metabolism), cell communication, signal transduction, energy, and cell growth/maintenance pathways constituted the most represented biological processes in which the identified mRNA transcripts are involved, therefore impacting in important cancer-promoting pathways, such as cell cycle regulation, mitosis, epithelial-mesenchymal transition (EMT), DNA repair, and telomere maintenance (Figure 4D). Of note, the expression of some of these transcripts positively correlated with worse disease severity (i.e., advanced tumor stage and tumor dedifferentiation) in the TCGA and Copenhagen cohorts (Supplementary Figure S3). Among all the 105 transcripts identified, *c-Maf inducing protein* (*CMIP*), *glutamate decarboxylase 1* (*GAD1*), *nucleoside diphosphate kinase 1* (*NME1*), *CDP-diacylglycerol synthase 1* (*CDS1*), and *cyclin-dependent kinases regulatory subunit 1* (*CKS1B*) constituted the best liquid biopsy candidate biomarkers, as their levels were increased in serum EVs and presented AUC values of 0.957, 0.928, 0.899, 0.893, and 0.891, respectively, for the diagnosis of CCA in comparison with patients with PSC, UC, and healthy individuals grouped together (Figure 5A–E). Importantly, a panel comprised of *CMIP*, *NME1* and *CKS1B* provided the maximum diagnostic capacity (AUC: 1.000) for CCA in comparison with the group containing healthy individuals and patients with PSC and UC (Figure 5F). The mRNA levels of the aforementioned transcripts were also markedly increased in serum EVs from patients with CCA compared with the ones isolated from PSC patients, therefore potentially constituting novel biomarkers for the differential diagnosis of CCA and PSC. The mRNA expression levels of these genes were also increased in CCA tumor samples from the TCGA and Copenhagen cohorts, compared with either normal surrounding liver tissue or normal intrahepatic bile ducts, being also found upregulated in CCA tumor cells, compared to NHCs, and also in CCA-derived EVs (Figure 5A–E). Noteworthy, besides providing the best diagnostic value among all the liquid biopsy candidates, *CMIP* mRNA levels were found particularly increased in poorly-differentiated tumors in the Copenhagen cohort (Supplementary Figure S3A).

### 3.4. Differential Transcriptomic Profiles of Urine EVs from Patients with CCA, PSC, UC, and Healthy Individuals

**Figure 6:**
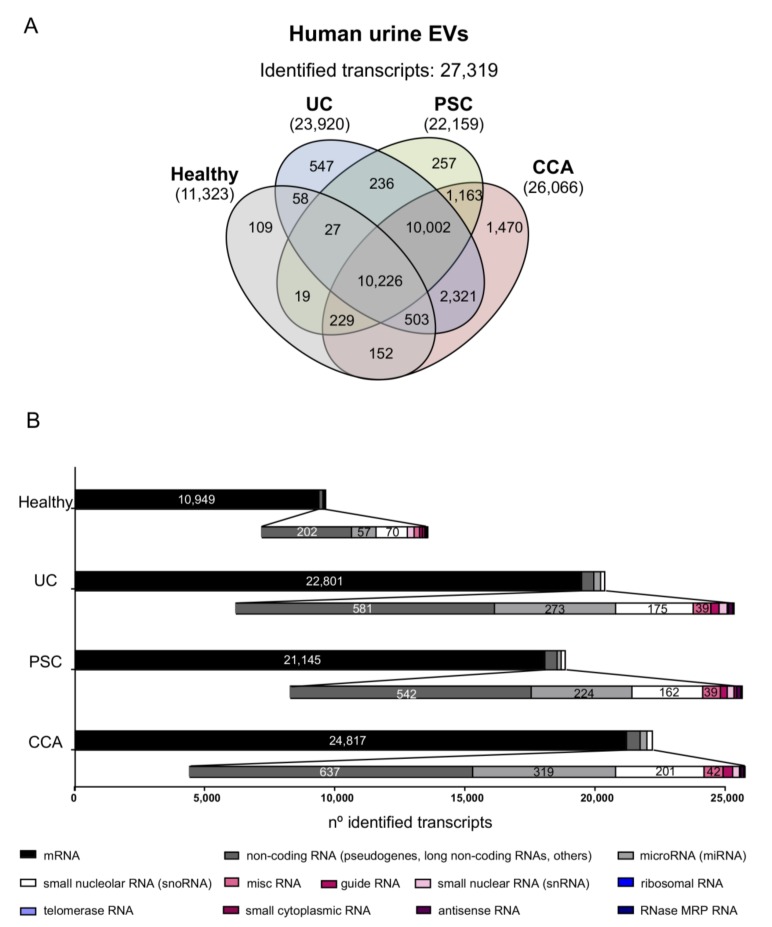
Comparative transcriptomic analysis of urine EVs from patients with CCA, PSC, or UC, and healthy individuals. (**A**) Venn diagrams showing the number of transcripts identified per group. Venn diagrams were generated using InteractiVenn web-based tool [29]. (**B**) Number of transcripts identified within each study group, subclassified according to their type [messenger RNA (mRNA), non-coding RNA (including mostly pseudogenes and long non-coding RNAs, among others), miscelaneous RNA (miscRNA), guide RNA, microRNA (miRNA), small nucleolar RNA (snoRNA), small nuclear RNA (snRNA), ribosomal RNA, telomerase RNA, small cytoplasmic RNA, antisense RNA and RNase MRP RNA]. In all groups, mRNAs constitute the most abundantly identified RNAs, followed by non-coding RNAs (pseudogenes, lncRNAs, and others), miRNAs, and snoRNAs.

**Figure 7:**
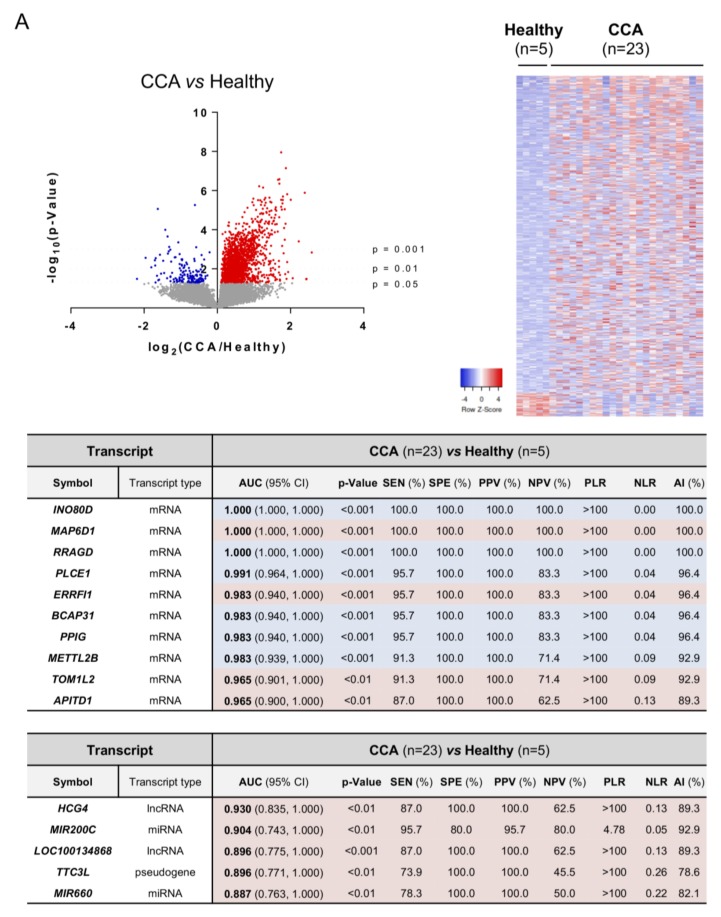

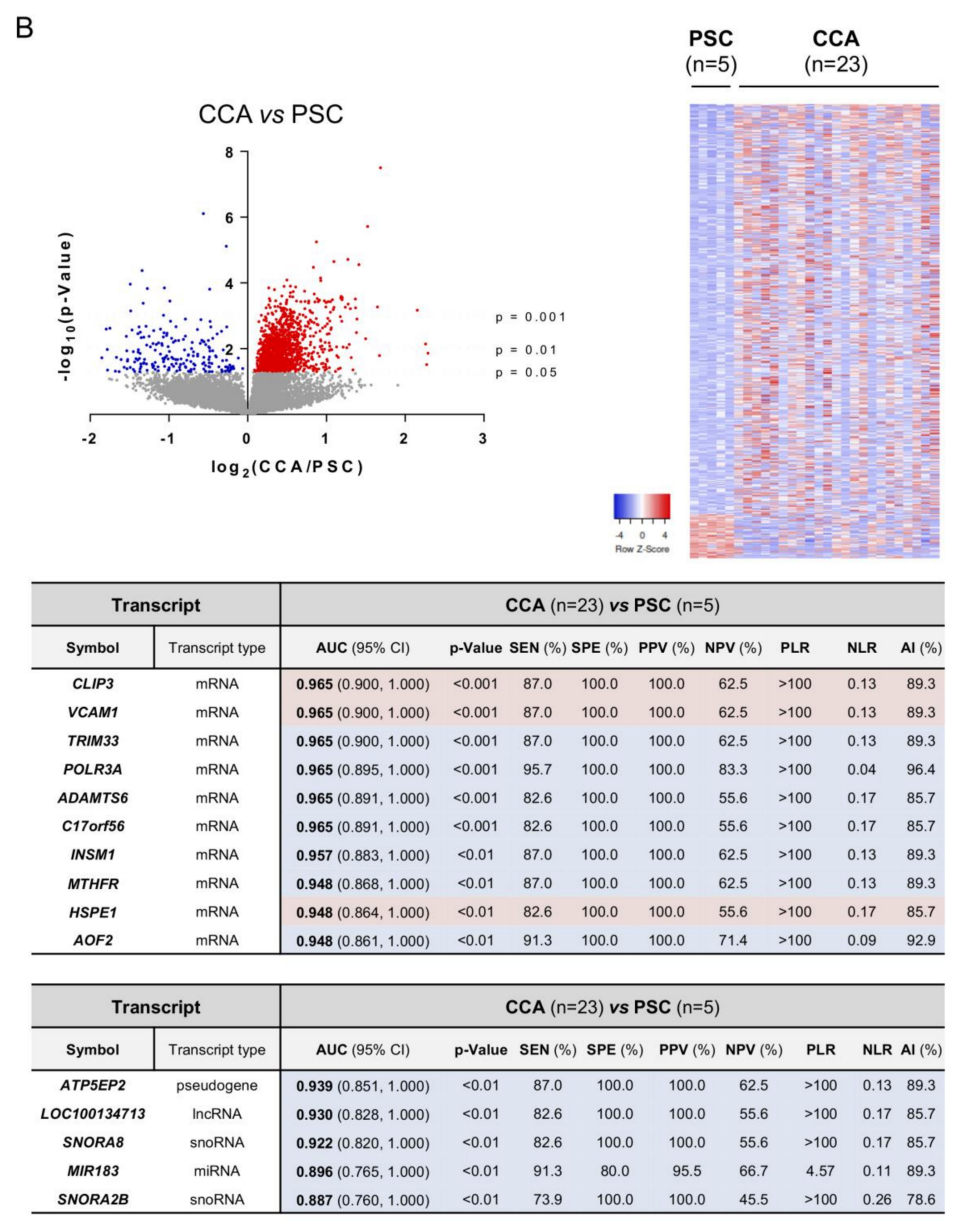

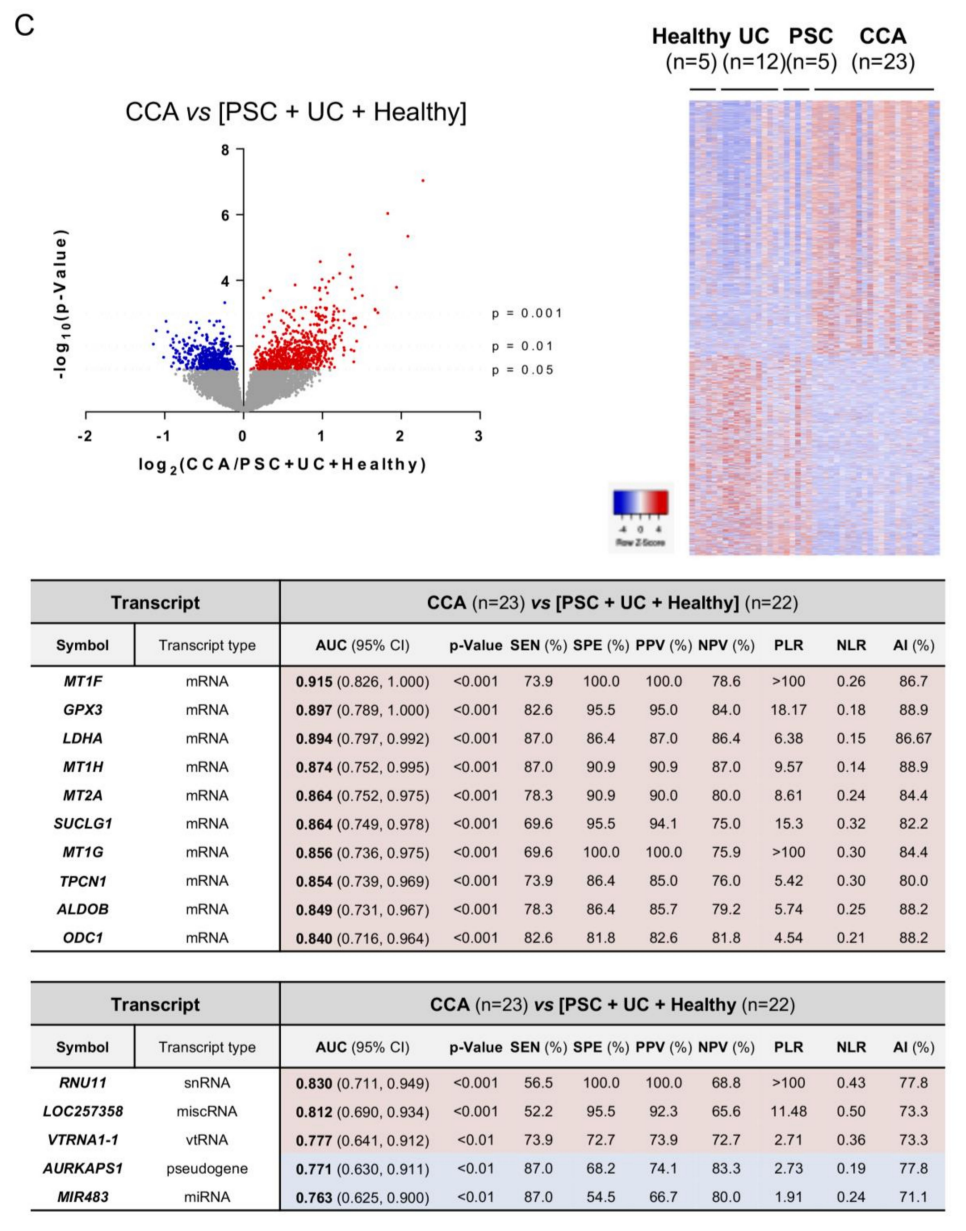
Differential transcriptomic profile of urine EVs and diagnostic capacity. Volcano plot (−log_10_(*p*-value) and log_2_(fold-change); up left), heatmap of the differentially expressed transcripts (up right) and diagrams with the diagnostic capacity with the highest AUC values of the 10 selected mRNAs and 5 selected non-coding RNAs in serum EVs from (**A**) CCA vs. Healthy individuals; (**B**) CCA vs. PSC; (**C**) CCA vs. (PSC + UC + Healthy individuals). Abbreviations: AI, accuracy index; AUC, area under the receiver operating characteristic curve; CI, confidence interval; miRNA, microRNA; lncRNA, long non-coding RNA; miscRNA, miscellaneous RNA; NLR, negative likelihood ratio; NPV, negative predictive value; PLR, positive likelihood ratio; PPV, positive predictive value; SEN, sensitivity; snRNA, small nuclear RNA; snoRNA, small nucleolar RNA; SPE, specificity; vtRNA, vault RNA.

In urine EVs, a total of 11,323, 23,920, 22,159, and 26,066 RNAs were identified in healthy individuals and patients with UC, PSC, or CCA, respectively, accounting for a total of 27,319 transcripts detected (Figure 6A). Regarding the type of transcripts found, similar distribution was observed when compared with the profile obtained in serum EVs, abundantly detecting mRNAs, followed by ncRNAs such as pseudogenes and lncRNAs, miRNAs, and snoRNAs (Figure 6B). The analysis of urine EVs revealed differential transcriptomic profiles between patients with CCA, PSC, or UC, and healthy individuals. In particular, 2386 transcripts were differentially identified in urine EVs from patients with CCA vs. healthy individuals, 1999 in CCA vs. PSC, and 1329 in CCA vs. other diseases (PSC and UC) and healthy individuals combined in a single group. In this regard, *INO80 complex subunit D* (*INO80D*), *MAP6 domain containing 1* (*MAP6D1*) and *Ras-related GTP binding D* (*RRAGD*) constituted the most promising mRNA biomarkers in urine EVs isolated from patients with CCA, in comparison with healthy individuals, displaying AUC values of 1.000. Of note, the lncRNA *HLA complex group 4* (*HCG4*), *miR200c*, and the lncRNA *LOC100134868* also presented high accuracies for the diagnosis of CCA, with AUC values of 0.930, 0.904, and 0.896, respectively (Figure 7A). Regarding the differential identification of CCA and PSC, the mRNA transcripts *CAP-Gly domain containing linker protein 3* (*CLIP3*), *Vascular cell adhesion molecule 1* (*VCAM1*) and *Tripartite motif containing 33* (*TRIM33*) displayed excellent AUC values (0.965), in parallel with the pseudogene *ATP synthase F1 subunit epsilon pseudogene 2* (*ATP5EP2*), the lncRNA *LOC100134713*, and the *Small nucleolar RNA, H/ACA box 8* (*SNORA8*) (AUC: 0.939, 0.930, and 0.922, respectively), thus allowing to distinguish patients with CCA and PSC with high sensitivity and specificity values (Figure 7B). When comparing urine EVs from patients with PSC vs. UC and healthy individuals considered as a unique group, several mRNA and non-coding RNAs arose as potential biomarkers for the diagnosis of PSC, with AUC values up to 1.000 and 0.965, respectively (Supplementary Figure S4). Finally, by comparing CCA to the group containing patients with other diseases (PSC and UC) and healthy individuals, the mRNA *metallothionein 1F* (*MT1F*), *glutathione peroxidase 3* (*GPX3*), and *lactate dehydrogenase 4* (*LDH4*) stood out as the ones with the best AUC values (0.915, 0.897, and 0.894, respectively), along with the ncRNAs *U11 small nuclear* (*RNU11*), *LOC257358*, and *vault RNA 1-1* (*VTRNA1-1*) (0.830, 0.812, and 0.777, respectively) (Figure 7C), therefore constituting promising urine EV biomarkers for the accurate diagnosis of CCA.

### 3.5. Selective mRNAs Present in Urine EVs from Patients with CCA Mimic Their Levels in Human Tumor Tissue, CCA Cells In Vitro, and EVs-Derived from Tumor Cholangiocytes

**Figure 8:**
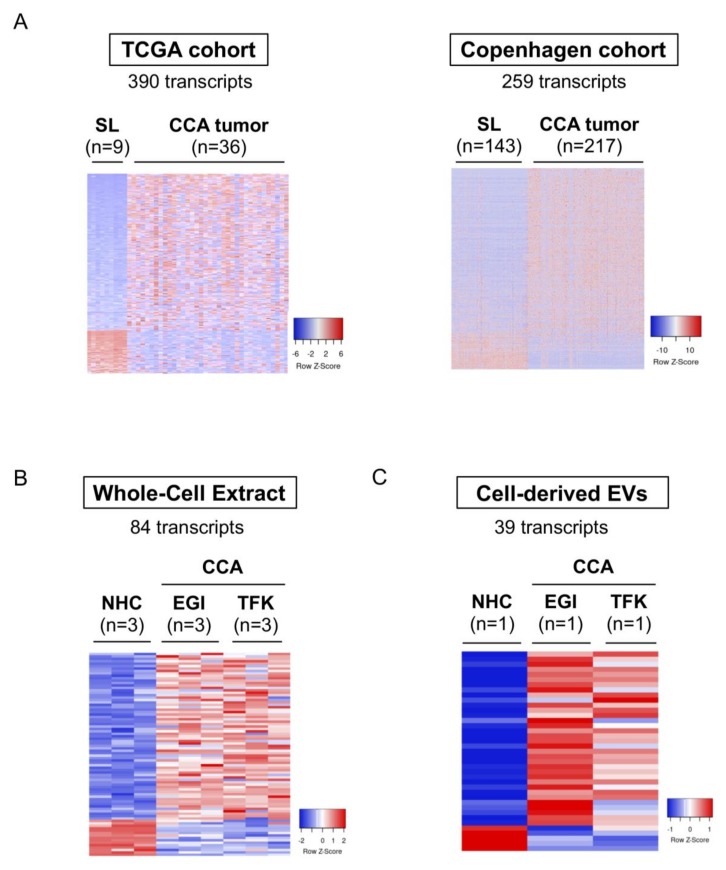

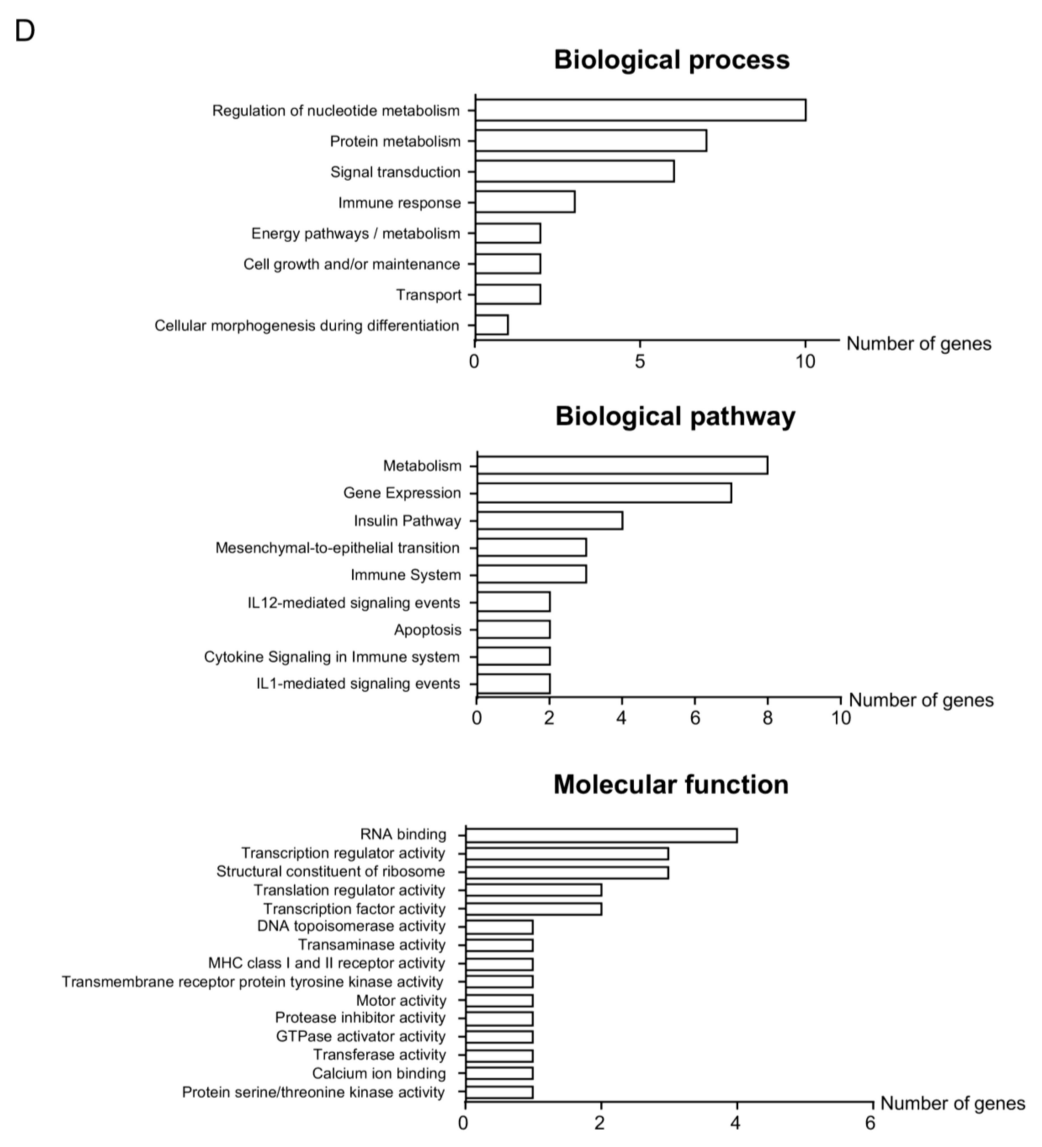
mRNAs commonly deregulated between urine EVs, CCA tumors from two independent cohorts of patients, tumor cells in vitro and in CCA-derived EVs. mRNAs differentially abundant in serum EVs from patients with CCA vs. (PSC + UC + Healthy individuals) were compared with the transcriptome of: (i) patients with CCA from The Cancer Genome Atlas TCGA (n = 36) and “Copenhagen” (n = 217) cohorts, (ii) CCA cells (EGI1 and TFK1) and cell-derived EVs compared to their respective control groups, further selecting the ones that are commonly expressed. Heatmap of the differentially expressed transcripts in (**A**) TCGA (left) and “Copenhagen” cohorts (right); (**B**) Whole-cell extracts from CCA cells and normal human cholangiocytes (NHCs); (**C**) Cell-derived EVs; (**D**) Gene ontology (GO: FunRich database [27]) analysis of the 105 transcripts commonly altered in serum EVs, CCA human tumors, CCA cells and in cell-derived EVs, highlighting the biological processes and pathways in which the identified transcripts are involved, as well as their biological function. Abbreviations: EVs, extracellular vesicles; NHC, normal human cholangiocyte; SL, surrounding liver; TCGA, The cancer genome atlas.

Similar to our previous analysis on the serum EV biomarkers identified in patients with CCA, compared to all the other study groups, we now selected the 1329 RNA transcripts that were significantly altered in urine EVs from patients with CCA and compared their expression levels with CCA and surrounding tumor tissues from both TCGA and Copenhagen cohorts. After performing a comprehensive analysis of these mRNAs in the TCGA cohort, we were able to identify 390 dysregulated transcripts (305 upregulated and 85 downregulated) that are commonly altered in urine EVs and in tumor samples from patients with CCA (Figure 8A, left). Additionally, compared to the changes observed in urine EVs, 259 transcripts also shared the same pattern of alteration in the Copenhagen cohort, with 206 transcripts presenting increased expression while 53 transcripts were reduced, when compared with surrounding liver (Figure 8A, right). The comparison of these 259 transcripts with the differential transcriptome of CCA cell lines compared to NHCs, revealed 84 shared mRNAs that were altered in CCA cells, with 69 being upregulated and 15 displaying decreased expression (Figure 8B). In EVs isolated from CCA and NHC cell cultures, we were able to identify 39 mRNAs (34 upregulated and 5 downregulated) commonly altered with urine EVs, tumor tissue, and CCA cells (Figure 8C).

**Figure 9:**
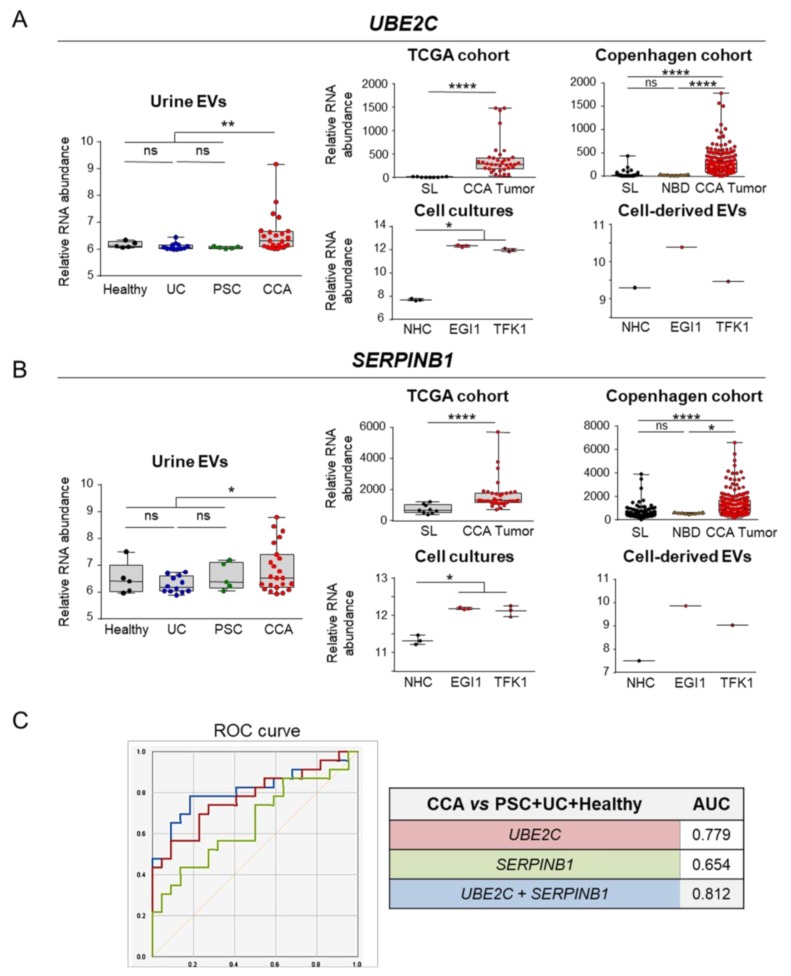
Potential urine liquid biopsy markers for CCA. From the 39 transcripts commonly found in serum EVs and differentially expressed in patient samples, CCA cells and in cell line-derived EVs, 2 potential urine liquid biopsy markers with the best diagnostic capacity were selected. Box plot diagrams with the mRNA transcript abundance in urine EVs (left) and the expression in the TCGA and “Copenhagen” cohorts, cholangiocyte cell lines and cell line-derived EVs (right) for (**A**) *Ubiquitin conjugatin enzyme E2 C* (*UBE2C*) and (**B**) *Serine proteinase inhibitor B1* (*SERPINB1*). (**C**) Diagnostic prediction (ROC curves and AUC values) of the selected urine liquid biopsy markers and from the combination of *UBE2C* and *SERPINB1* for the diagnosis of CCA in comparison with (PSC + UC + Healthy individuals). Abbreviations: AUC, area under the receiver operating characteristic curve; EVs, extracellular vesicles; NHC, normal human cholangiocyte; NBD, normal bile ducts; SL, surrounding liver; TCGA, The cancer genome atlas.

In order to evaluate the role of these transcripts in carcinogenesis, we conducted a GO analysis and observed that, in resemblance with what we previously found in serum EVs, these mRNA transcripts code for proteins that are predominantly related with tumor development and progression, namely metabolic pathways (nucleic acids and protein metabolism), signal transduction, cell communication, EMT and immune response. Although less represented, some transcripts were also linked to energy and cell growth/maintenance pathways (Figure 8D). In this regard, the transcripts *ubiquitin conjugating enzyme E2 C* (*UBE2C*) and *serine protease inhibitor B1* (*SERPINB1*) arose as potential liquid biopsy biomarkers, being increased in urine EVs isolated from patients with CCA in comparison to a group containing patients with PSC, UC and healthy controls (Figure 9A,B). Combining these urine biomarkers into one panel increased their diagnostic accuracy, providing an AUC value of 0.812 for the diagnosis of CCA (Figure 9C). Noteworthy, the expression levels of these transcripts were also markedly upregulated in CCA tumor samples from the TCGA and Copenhagen cohorts, when compared with both normal surrounding liver specimens and/or normal intrahepatic bile ducts, presenting also increased expression in CCA cells and in CCA-derived EVs, when compared with NHCs (Figure 9A,B). Importantly, *SERPINB1* mRNA levels increased with disease severity in the Copenhagen cohort, being particularly overexpressed in advanced tumor stages compared with early stage CCAs (Supplementary Figure S5A). Furthermore, although not being presented as one of the best liquid biopsy candidate, *Tctex1 domain containing 2* (*TCTEX1D2)* levels were found upregulated in poorly-differentiated tumors compared with well-differentiated ones (Supplementary Figure S5B).

## 4. Discussion

In the last decade, a considerable effort has been made to identify novel non-invasive biomarkers for the early and accurate diagnosis of CCA [7]. Here, we report for the first time the differential RNA profile of serum and urine EVs from patients with CCA, PSC, or UC, and healthy individuals, identifying new potential biomarkers with high diagnostic capacity. Noteworthy, some of the altered mRNAs were similarly changed in CCA tumors from two independent cohorts of patients, and in tumor cells and CCA-derived EVs in vitro, highlighting their utility as liquid biopsy biomarkers as well as their potential value as targets for therapy.

High-throughput omic approaches have been of great help in order to find potential new candidate biomarkers. In fact, the identification of the proteomic content of EVs, as well as certain circulating proteins, in biofluids have already provided candidate biomarkers in bile, serum, and urine from patients with CCA [18,30,31,32]. We have recently described the differential proteomic profiles of serum EVs from patients with CCA, compared to HCC, PSC, and healthy individuals, reporting new potential protein biomarkers with high diagnostic capacity [18] that must be internationally validated by ELISA technology. Nevertheless, a full transcriptomic analysis in distinct body fluids (in particular EVs) from these patients has never been conducted and might result in the identification of novel, accurate biomarkers for the diagnosis of CCA. Although proteins are usually more stable than mRNAs, their presence within EVs provides them protection from degradation; moreover, RNAs are usually easier to detect and quantify, even when found at very low levels, which may help in their faster translation into the clinic [33]. In fact, circulating small ncRNAs are found in all biofluids (including serum and urine), mainly due to their remarkable resistance to RNase degradation. High circulating RNase levels contribute to a low abundance of other types of RNAs (mRNAs) and significantly compromise their easy and reliable detection [34]. Still, specific RNAs can be released from cancer cells into biofluids, allowing their identification and further determination of their potential value as biomarkers, as they may mirror the cellular state within the concept of liquid biopsy. For instance, some RNA transcripts were already evidenced and found increased in plasma, as is the case of *telomerase reverse transcriptase* (*hTERT*) that showed diagnostic and prognostic value for prostate cancer, being a good predictor of recurrence [35,36]. Similarly, the levels of the long non-coding RNA *prostate cancer associated 3* (*PCA3*) were abundantly found in urine of patients with prostate cancer, constituting a promising non-invasive biomarker for the diagnosis of that cancer [37,38]. The levels of several miRNAs were also reported altered in serum, plasma, and urine of patients with gastrointestinal cancers, including CCA, constituting also potential novel biomarkers for cancer diagnosis [7,33,39,40,41].

**Figure 10:**
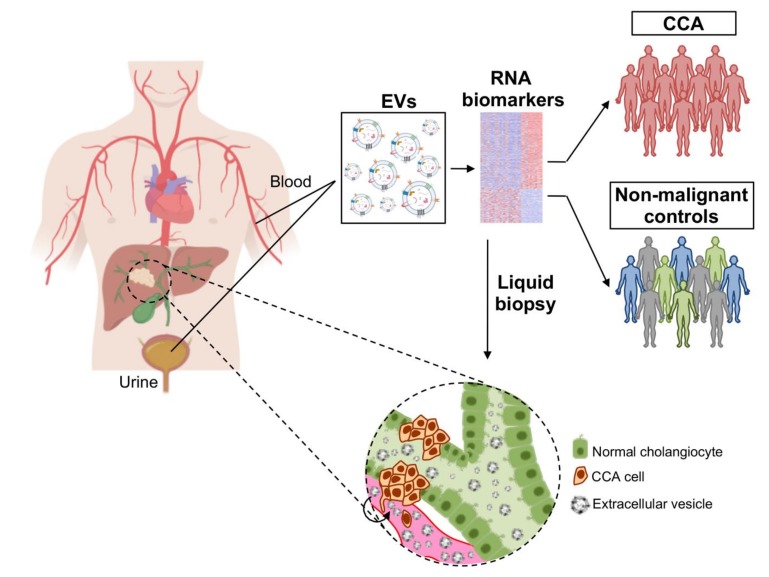
Novel liquid biopsy approach for cholangiocarcinoma. CCA tumor cells display distinct RNA expression profiles which are later released into circulation in EVs, containing potential biomarkers for CCA, that are amenable for detection in serum and urine, thus constituting a novel liquid biopsy approach.

Taking advantage from our previously reported EV isolation protocol [18], we have here also settled up the protocol for the isolation of urine EVs. By analyzing the transcriptomic profile of serum and urine EVs from patients with CCA, the present study was pioneer in identifying novel potential RNA biomarkers with high diagnostic capacity for CCA. Noteworthy, some of these new RNA biomarkers were also significantly altered in CCA tissue from the two international cohorts of patients and further disturbed in CCA cell lines and in EVs secreted from these tumor cell lines, when compared with NHCs. Consequently, these biomarkers are mirroring what is happening in the tumor tissue, since the disturbances observed in tumor biopsies (and CCA cell lines) that are later secreted in EVs and released into the bloodstream are amenable for detection either in serum or urine (Figure 10). This constitutes a novel and innovative liquid biopsy approach where we are able to detect specific alterations that are observed in tumor tissue without obtaining tumor samples, harboring a high diagnostic value. In the future, evaluating the relevance of these biomarkers in predicting prognosis and in guiding therapeutic decisions is envisioned.

Taking into consideration the 105 potential liquid biopsy biomarkers that were identified in serum, we herein reported the best five serum biomarkers that display excellent diagnostic accuracy: *CMIP*, *GAD1*, *NME1*, *CDS1*, and *CKS1B*. Importantly, these novel potential biomarkers might constitute better CCA biomarkers than CA19-9 since the AUC values that we herein obtained (up to 0.891) are higher than the diagnostic capacity reported in a systematic review and a meta-analysis, in which the AUC value for CA19-9 was 0.830 [42]. Despite the potential diagnostic value of these transcripts, considering that they are concomitantly increased in tumor tissue in two international independent cohorts, we postulate that they also might play a pivotal pathological role during cholangiocarcinogenesis. Still, no studies have currently addressed the involvement of these biomolecules in CCA, although several works already valued their role in other types of cancer. For instance, the transcription factor CMIP was previously shown to be increased in human gastric cancer and glioma tumors, contributing to tumor proliferation and metastasis [43,44]. Furthermore, high CMIP levels were associated with worse prognosis (recurrence-free and overall survival) in gastric and breast cancers [43,45] and were related with herceptin resistance in HER2-positive gastric cancer cells [46]. Similarly, the enzyme encoded by *GAD1* gene, which catalyzes the conversion of L-glutamic acid to γ-aminobutyric acid, has been found overexpressed in several types of tumors, including lung adenocarcinoma [47], nasopharyngeal carcinoma [48], oral squamous cell carcinoma [49], prostate cancer [50], and brain metastasis [51], being also found upregulated in colon and HCC cells in vitro [52]. In parallel, high GAD1 levels were also shown to correlate with the pathological stage of patients with lung adenocarcinoma, positively correlating with metastasis and with worse recurrence-free survival [47]. Regarding *NME1*, its relevance in cancer is still controversial. A meta-analysis evaluated the prognostic value of NME1 in patients with digestive system neoplasms (including patients with HCC and gallbladder cancer, but not CCA) and reported that high NME1 levels are correlated with well-differentiated tumors and with less-severe cancer stages, with no evident correlation with prognosis [53]. In agreement with the reported metastasis-suppressing role of NME1, patients with HCC presenting lower protein levels of NME1 displayed increased metastatization [54]. In patients with HCC [55] and in two animal models of HCC [56], *NME1* expression was found upregulated in comparison with non-tumor tissue, while knocking-out *NME1* resulted in an increased number of lung metastasis [56]. Still, increased levels of NME1 negatively correlated with disease stage while NME1 upregulation was positively correlated with poor overall survival and with higher recurrence [55]. Furthermore, in melanoma patients, a *NME1*-related gene expression signature was reported and linked with increased overall survival [57], pinpointing for a potential tumor suppressor role, but new pro-oncogenic roles, mainly related with the expansion of stem-like features and tumor growth, were recently reported [58]. Considering *CDS1*, a DNA damage-related function is well-known [59,60] and a screening of several cancer cell lines highlighted that the expression levels of CDS1 are dependent on p53 activity, being inversely correlated with the presence of a functional p53 [61]. Although CDS1 is thought to act as a tumor suppressor, there is no much information in this field. For instance, CDS1 was shown to be decreased in patients with HCC, likely as a result of promoter hypermethylation [62]. Nevertheless, its relevance for hepato- and/or cholangiocarcinogenesis is yet to be unveiled. Additionally, *CKS1B* represents an oncogene that has been reported increased in retinoblastoma, HCC, nasopharyngeal carcinoma and multiple myeloma [63,64,65,66,67]. Importantly, *CKS1B* upregulation has been positively related to increased proliferation, migration, angiogenesis, invasion and chemoresistance, being further related with lymph node metastasis and worse prognosis. Overall, regarding these genes, although nothing is still reported in CCA pathogenesis, they potentially constitute new key carcinogenic players that deserve further attention in the near future.

Considering the potential urine liquid biopsy biomarkers, we herein were able to report 39 transcripts that are differentially found in EVs from patients with CCA, sharing their pattern of expression in CCA patients from the TCGA and Copenhagen cohorts and in CCA cells and in CCA-derived EVs in vitro. In fact, this number of transcripts corresponds to almost one-third of the identified serum liquid biopsy biomarkers, which might indicate that serum constitutes a richer source of potential CCA biomarkers. In this line, we have to bear in mind that EVs released from tumor masses directly enter into the bloodstream and their analysis might better mirror what is currently happening in the tumor. Furthermore, glomerular filtration might impact on the detected tumor-released EVs, therefore explaining the reduced number of urine liquid biopsy biomarkers. Still, urine analysis is considered the less invasive procedure for the diagnosis of cancer so far and we here describe some potential new urine biomarkers with good diagnostic values that are also altered in tumor samples. For instance, *UBE2C* is commonly upregulated in intestinal-type gastric cancer, ovarian cancer, head and neck squamous cell carcinoma, pancreatic ductal adenocarcinoma, HCC, and non-small cell lung cancer [68,69,70,71,72,73,74]. Remarkably, increased *UBE2C* expression promotes chromosomal instability, cell cycle progression, proliferation and EMT, being positively correlated with worse clinical outcomes, mainly overall survival, lymph node metastasis, and progression-free survival. Of note, experimental inhibition of *UBE2C* suppressed the malignant phenotypes, surpassing cisplatin resistance in ovarian and non-small cell lung cancers [69,74], in parallel with overcoming sorafenib resistance in HCC [73]. On the other hand, the reports regarding *SERPINB1* are still controversial. SERPINB1 levels were found decreased in patients with prostate cancer, glioma, and HCC, favoring migration and invasion [75,76,77], while displaying increased expression in cells and patients with oral cancer, correlating with high motility and cell migration in vitro [78]. Importantly, SERPINB1 levels were shown to predict the outcome of cisplatin-based chemotherapies in melanoma [79] and the value of this transcript/protein as surrogate marker for CCA should be evaluated in the future. 

Besides identifying novel CCA biomarkers, we were also able to identify novel potential RNA transcripts for the differential diagnosis of CCA vs. PSC. Distinguishing between PSC-associated benign biliary strictures and early-stage CCA lesions is challenging. In fact, magnetic resonance imaging (MRI) has a limited resolution for this differential diagnosis [5,80] and performing conventional cytology and/or fluorescence in situ hybridization (FISH) after invasive biliary brushing by endoscopic retrograde cholangiography only display a moderate diagnostic accuracy and increase the odds for procedure-related complications, including pancreatitis and cholangitis [5,80]. In fact, several serum and urine EV transcripts provided the maximum diagnostic capacity for the differential diagnosis of CCA and PSC, constituting potential biomarkers for the early diagnosis of CCA in patients with PSC. Although this work was mainly focused on the study of mRNA transcripts, several ncRNAs stood out as novel potential diagnostic biomarkers for the diagnosis of CCA. In particular, the levels of the lncRNA *MALAT1* are increased in serum EVs from patients with CCA compared to PSC, displaying an AUC value of 1.00. In this regard, *MALAT1* was found upregulated in HCC, contributing to tumor development and progression [81,82,83,84], as well as identified in HCC-derived exosomes [85]. *MALAT1* is also increased in colon cancer cells, directly binding to miR-663a, thus acting as a competing endogenous lncRNA; as a result, important cancer-related miR-663a targets (*TGFB1, PIK3CD, P21*, *JunB*, and *JunD*) were affected [86]. These results encourage future studies on the potential role of MALAT1 in CCA. In addition, increased levels of miR-604 and miR-551B in serum EVs from patients with CCA also provided excellent diagnostic capacities (up to 0.944) for CCA and some studies already started to evaluate their involvement in carcinogenesis and as diagnostic biomarkers [87,88,89,90]. Considering the high resistance of small ncRNAs to RNase degradation, additional efforts should be employed in order to validate their diagnostic accuracy. 

Overall, here we reported for the first time the differential RNA profiles of serum and urine EVs of patients with CCA, PSC, or UC, compared to healthy individuals, identifying novel non-invasive accurate biomarkers for CCA that mirror their expression in tumor tissue, thus constituting a novel liquid biopsy approach. The newly identified RNA biomarkers might markedly facilitate the diagnosis of these diseases and should be taken into consideration in future studies. These results pave the path not only for the discovery of new biomarkers but also for new potential therapeutic targets, as they may participate in disease pathogenesis. However, these results need to be now validated in large, international, and well-characterized cohorts of patients (and also including patients with CCA on a PSC background), in order to ascertain the diagnostic accuracy of the proposed biomarkers and then translate our findings into clinics. More importantly, it is pivotal to validate these results with easy transferable techniques [e.g., quantitative PCR (qPCR) and droplet digital PCR (ddPCR), among others], in order to avoid the time-consuming steps and equipment demands for EVs isolation. In this way, we will be a little bit closer to see the use of accurate non-invasive diagnostic biomarkers for CCA at the clinics. 

## Supplementary Materials

The following are available online at https://www.mdpi.com/2073-4409/9/3/721/s1, Figure S1: Differential transcriptomic profile and diagnostic efficacy of serum EV transcripts; Figure S2: Characterization of cholangiocyte-derived EVs transcriptome. Figure S3: Comparative analysis of the levels of candidate serum liquid biopsy transcripts with CCA disease severity in the Copenhagen and TCGA cohorts; Figure S4: Differential transcriptomic profile and diagnostic efficacy of urine EV transcripts; Figure S5: Comparative analysis of the levels of candidate urine liquid biopsy transcripts with CCA disease severity in the Copenhagen and TCGA cohorts; Table S1: Clinical information of the individuals; Table S2: Number of transcripts identified in each experimental group.

## Abbreviations

AI: accuracy index; ANOVA, analysis of variance; ATF4, activating transcription factor 4; ATP5EP2, ATP synthase F1 subunit epsilon pseudogene 2; AUC, area under de receiver operating curve; CA19-9, carbohydrate antigen 19-9; CCA, cholangiocarcinoma; CDS1, CDP-diacylglycerol synthase 1; CEA, carcinoembryonic antigen; CLIP3, CAP-Gly domain containing linker protein 3; CMIP, c-MAF inducing protein; CT, computed tomography; dCCA, distal CCA; ddPCR, droplet digital PCR; CKS1B, CDC28 protein kinase regulatory subunit 1B; DTT, dithiothreitol; ERCP, endoscopic retrograde cholangiopancreatography; EVs, extracellular vesicles; FAM107B, family with sequence similarity 107 member B; FBS, fetal bovine serum; GAD1, glutamate decarboxylase 1; GO, gene ontology; GPX3, glutathione peroxidase 3; Grp78, 78 kDa glucose-regulated protein; HCC, hepatocellular carcinoma; HCG4, HLA complex group 4; H&E, hematoxylin and eosin; hTERT, telomerase reverse transcriptase; IBD, inflammatory bowel disease; iCCA, intrahepatic CCA; INO80D, INO80 complex subunit D; LDH4, lactate dehydrogenase 4; MALAT1, metastasis associated lung adenocarcinoma transcript 1; MAP6D1, MAP6 domain containing 1; miRNA, microRNA; MRCP, magnetic resonance cholangiopancreatography; MRI, magnetic resonance imaging; MT1F, metallothionein 1F; MVs, microvesicles; NHCs, normal human cholangiocytes; NLR, negative likelihood ratio; NME, NME/NM23 nucleoside diphosphate kinase 1; NPV, negative predictive value; NTA, nanoparticle tracking analysis; pCCA, perihilar CCA; OR4F3, olfactory receptor family 4 subfamily F member 3; PBS, phosphate-buffered saline; PCA3, prostate cancer associated 3; PET, positron emission tomography; PFA, paraformaldehyde; PHGDH, phosphoglycerate dehydrogenase; PLR, positive likelihood ratio; PMS2L4, PMS1 homolog 2 mismatch repair system component pseudogene 4; PON1, paraoxonase 1; PPV, positive predictive value; P/S, penicillin/streptomycin; PSC, primary sclerosing cholangitis; PTC, percutaneous transhepatic cholangiography; qPCR, quantitative PCR; RFFL, ring finger and FYVE like domain containing E3 ubiquitin protein ligase; RRAGD, Ras related GTP binding D; SDS-PAGE, sodium dodecyl sulfate–polyacrylamide gel electrophoresis; SEN, sensitivity; SERPINB1, serpin family B member 1; SNORA8, small nucleolar RNA, H/ACA box 8; SPE, specificity; TBS, tris-buffered saline; TCGA, the cancer genome atlas; TCTEX1D2, Tctex1 domain containing 2; TEM, transmission electron microscopy; TRIM33, tripartite motif containing 33; UA, uranyl acetate; UBE2C, ubiquitin conjugating enzyme E2 C; UC, ulcerative colitis; VCAM1, vascular cell adhesion molecule 1; VTRNA1-1, vault RNA1-1; WCEs, whole-cell extracts; ZNF266, zinc finger protein 266.
